# *Cubilin* gene mutation screening in Thai Border Collies using double-mismatch allele-specific and multiplex polymerase chain reaction: Implications for Imerslund–Gräsbeck syndrome

**DOI:** 10.14202/vetworld.2026.580-590

**Published:** 2026-02-17

**Authors:** Chommanad Lerdkrai, Nattaphong Akrimajirachoote, Nuch Phungphosop

**Affiliations:** Department of Physiology, Faculty of Veterinary Medicine, Kasetsart University, Bangkok, Thailand

**Keywords:** Border Collies, *cubilin* gene, double-mismatch allele-specific PCR, genetic screening, Imerslund–Gräsbeck syndrome, multiplex PCR, Thailand, veterinary genetics

## Abstract

**Background and Aim:**

Imerslund–Gräsbeck syndrome (IGS) is a rare autosomal recessive disorder characterized by selective intestinal cobalamin malabsorption in humans and several canine breeds. In Border Collies, IGS is caused by a single cytosine deletion in exon 53 of the *CUBN* gene (c.8392delC), resulting in a frameshift mutation and loss of Cubam receptor function. Although carrier frequencies have been reported in European and East Asian populations, region-specific genetic epidemiology data from Southeast Asia are lacking. This study aimed to determine the genotypic distribution and allele frequency of the *CUBN* c.8392delC mutation in Thai Border Collies and to validate a cost-effective molecular diagnostic approach combining double-mismatch allele-specific primers with multiplex polymerase chain reaction (PCR).

**Materials and Methods:**

A total of 107 clinically healthy Border Collies from private owners and breeding kennels in Thailand were enrolled. Genomic DNA extracted from peripheral blood was genotyped using a newly optimized double-mismatch allele-specific multiplex PCR assay designed to discriminate wild-type and mutant *CUBN* alleles, with an internal control amplicon included in each reaction. Synthetic mutant DNA fragments were used as positive controls for homozygous mutant detection. All PCR-based genotyping results were independently validated by Sanger sequencing. Genotype and allele frequencies were calculated, and 95% confidence interval (CI) values were estimated using the Wilson score method.

**Results:**

Of the 107 dogs examined, 106 were homozygous wild-type (WT; *CUBN* +/+) (99.1%; 95% CI: 94.8%–99.8%), one dog was a heterozygous carrier (*CUBN* +/−) (0.9%; 95% CI: 0.16–5.0%), and no homozygous mutant individuals (*CUBN* −/−) were identified (0%; 95% CI: 0%–4.3%). The estimated wild-type and mutant allele frequencies were 99.6% (95% CI: 98.2%–99.9%) and 0.4% (95% CI: 0.07%–1.8%), respectively. The double-mismatch allele-specific multiplex PCR assay demonstrated 100% concordance with Sanger sequencing, with no false-positive or false-negative results.

**Conclusion:**

This study provides the first population-level screening data for the *CUBN* c.8392delC mutation associated with IGS in Thai Border Collies and indicates a very low carrier and mutant allele frequency in this genetically diverse population. The validated double-mismatch allele-specific multiplex PCR assay offers a rapid, reliable, and cost-effective tool for routine genetic screening, supporting evidence-based breeding strategies and future large-scale genetic surveillance of hereditary disorders in dogs.

## INTRODUCTION

Cobalamin (B12) is an essential water-soluble vitamin involved in multiple physiological processes, including hematopoiesis, neural transmission [1], nucleic acid synthesis, mitochondrial metabolism, intestinal microbial regulation [2], and antioxidant defense [3]. It functions as a cofactor for enzymes responsible for methylmalonic acid degradation and methionine synthesis [4]. In most carnivores, cobalamin is obtained predominantly from animal-derived dietary sources [5]. During digestion, dietary cobalamin initially binds to haptocorrin and is subsequently released by pancreatic enzymes in the intestine, enabling its interaction with the Cubam receptor located on the apical surface of ileal enterocytes. Following cellular uptake, cobalamin binds to transcobalamin, which mediates its transport throughout the systemic circulation [6, 7].

Cubam is a membrane-bound endocytic receptor expressed primarily in the terminal ileum, where it plays a central role in intestinal cobalamin absorption [6]. It is also expressed in the renal proximal tubule and participates in the reabsorption of filtered proteins, particularly albumin [8]. Cubam is a multimeric receptor complex composed of two subunits, amnionless (*AMN*) and cubilin (*CUBN*), encoded by the *AMN* and *CUBN* genes, respectively [6, 7]. Mutations in either gene have been associated with IGS in humans, following an autosomal recessive mode of inheritance [9, 10]. A comparable hereditary disorder has also been reported in several dog breeds, including Australian Shepherds [11, 12], Giant Schnauzers [13], Beagles [14–17], and Border Collies [18–21]. He *et al*. [11] identified two independent *AMN* gene mutations underlying IGS in Australian Shepherds and Giant Schnauzers. In contrast, IGS in Beagles and Border Collies has been linked to two distinct mutations in the *CUBN* gene [19, 20, 22].

In 2013, Fyfe *et al*. [19] and Owczarek-Lipska *et al*. [20] independently employed genomic sequencing to identify the causative mutation of IGS in Border Collies. Both studies reported a single cytosine deletion (CUBN:c.8392delC) in exon 53 of the *CUBN* gene on chromosome 2. This deletion induces a frameshift mutation, generating a premature stop codon in *CUBN* and leading to the production of a non-functional Cubam receptor. Consequently, intestinal cobalamin absorption is impaired, resulting in cobalamin deficiency and associated clinical manifestations, including lethargy, poor weight gain, anemia, hypocobalaminemia, hypoproteinemia, elevated liver enzyme activities, and hyperammonemia [16, 19–21]. Previous genetic surveys conducted in Switzerland [20] and Japan [23] reported IGS carrier frequencies of 6.2% and 3%, respectively.

Despite the established association between *CUBN* and *AMN* mutations and IGS in several canine populations, region-specific genetic epidemiology data for IGS in Thai Border Collies remain unavailable. Existing prevalence estimates are largely derived from European and East Asian cohorts, which differ substantially in population structure, breeding practices, and founder effects. Moreover, routine screening in Thailand is constrained by limited access to high-throughput or sequencing-based diagnostics, underscoring the need for a reliable, cost-effective, and scalable genotyping approach that can be implemented in clinical and breeding settings. Consequently, the distribution of *CUBN* variants and the true carrier frequency of IGS in Thai Border Collies have not yet been systematically characterized.

The aim of this study was to determine the genotypic distribution and allele frequency of the *CUBN* c.8392delC mutation associated with IGS in a cohort of Thai Border Collies using a double-mismatch allele-specific multiplex polymerase chain reaction (PCR) assay. In addition, this study sought to validate the accuracy and reliability of this assay against Sanger sequencing and to provide foundational data to support evidence-based genetic counseling, preventive breeding strategies, and future large-scale surveillance of hereditary disorders in Thai Border Collies.

## MATERIALS AND METHODS

### Ethical approval

The study was conducted in full compliance with national and institutional guidelines for the care and use of research animals. All procedures involving animals were reviewed and approved by the Institutional Animal Care and Use Committee (IACUC), Faculty of Veterinary Medicine, Kasetsart University, Thailand (Approval No. ACKU67-VET-064). The protocol adhered to the ethical principles outlined in the Guide for the Care and Use of Laboratory Animals, the Animal Welfare Act of Thailand, and the Animal Research: Reporting of In vivo Experiments 2.0 guidelines for reporting animal research.

Blood sampling was minimally invasive and performed exclusively by licensed veterinarians. No sedation or anesthesia was required for routine venipuncture; however, appropriate gentle manual restraint was used to minimize stress and ensure animal safety. Each owner (private and kennel owners) provided written informed consent for the collection of blood samples and the use of the animals’ genetic information strictly for research purposes. All samples were handled according to standard biosafety and biosecurity practices. Owner details and kennel identifiers were kept confidential, and no person or identifying information is disclosed in the publication. The IACUC verified that the study posed minimal risk, involved non-terminal and non-invasive sampling, and met all ethical requirements for genetic screening in companion animals.

### Study period and location

The research project was conducted from June 2024 to March 2025. All experiments were performed in the molecular research laboratory of the Department of Physiology, Faculty of Veterinary Medicine, Kasetsart University, Thailand (13.8446° N, 100.5773° E). Throughout all laboratory procedures, the room temperature was maintained at 22–25°C, and to ensure consistency, standard laboratory equipment was calibrated regularly. To minimize variability, the timing of each experimental step was strictly followed according to the validated protocol.

### Animals and sample collection

Border Collies were selected based on phenotypic characteristics defined in the Fédération Cynologique Internationale breed standard No. 297 to ensure breed authenticity. All dogs had an unknown *CUBN* genotype before genetic testing. The dogs included in this study were clinically healthy, aged between 3 months and 10 years, and had no history of inherited metabolic or hematological disorders. Both male and female dogs were included in this study.

A total of 107 dogs, comprising 33 privately owned dogs and 74 dogs from seven breeding kennels, representing both pedigree and non-pedigree backgrounds, were enrolled. Participation was voluntary, and owners and kennel managers provided informed consent for sample collection (Table 1). Pedigree information for dogs originating from registered breeding kennels was verified using breeder- and kennel-provided records, while pedigree data for privately owned dogs were obtained from owner-provided documentation when available.

**Table 1:** Distribution of dogs by source, sex, and age in the study population.

Source of dogs	Total (n)	Male, n (%)	Female, n (%)	Age range (months to years)
Privately owned dogs	33	18 (54.55)	15 (45.45)	3 months to 10 years
Kennel A	49	30 (61.22)	19 (38.78)	3 months to 7 years
Kennel B	5	3 (60)	2 (40)	3–7 months
Kennel C	8	3 (37.5)	5 (62.5)	6 months to 5 years
Kennel D	4	0 (0)	4 (100)	10 months to 2 years
Kennel E	2	2 (100)	0 (0)	4–5 years
Kennel F	2	0 (0)	2 (100)	5–6 months
Kennel G	4	1 (25)	3 (75)	4 months to 3 years
Total	107	57 (53.27)	50 (46.73)	–

Peripheral blood samples (1.5 mL per dog) were collected using minimally invasive venipuncture with gentle manual restraint. Samples were collected in tubes containing ethylenediaminetetraacetic acid and stored at 4°C until genomic DNA extraction.

### DNA extraction

Genomic DNA extraction was performed under controlled environment conditions (22–25°C) using the QIAamp DNA Blood Mini Kit (Qiagen GmbH, Hilden, Germany) according to the manufacturer’s instructions. The concentration and purity of DNA were evaluated using a NanoDrop™ 2000 spectrophotometer (Thermo Fisher Scientific, Waltham, MA, USA), with a typical concentration of 50–80 ng/µL and acceptable A260/A280 ratios between 1.8 and 2.0.

Samples with concentrations below the minimum concentration threshold and a low A260/A280 ratio were re-extracted and subsequently evaluated by agarose gel electrophoresis to confirm DNA integrity. High-quality DNA samples were aliquoted and stored at −20°C for long-term preservation, and stability was maintained for up to 12–18 months without detectable degradation.

### Primer design

This study developed a new set of primers using the integration of double-mismatch allele-specific primers with a multiplex PCR assay. Targeted sequences were identified using the Primer-BLAST tool (NCBI). All primers were designed using sequences deposited in GenBank under accession No. NC_006584.3.

In silico analysis of primer secondary structures, BLAST alignment results, and potential intermolecular interactions, including hairpin loops, self-dimers, and primer-dimer formation, were performed using the OligoAnalyzer™ Tool (Integrated DNA Technologies, Coralville, IA, USA). To ensure adequate purity for downstream PCR applications, oligonucleotides were synthesized and purified using the high-affinity purification method (Biobasic Inc., Markham, ON, Canada) (Table 2).

**Table 2:** Primer sequences used for *CUBN* genotype identification.

Primer name	Nucleotide sequence (5′–3′)	Tm (°C)	GC (%)	Product
Forward-Internal	AGCTCACTTACAACTCGGAGC	60.07	52.38	Internal control amplicon (611–612 bp)
Reverse-Common	TATGCCATGCCCTTTTGCCTA	60.06	47.62	—
Forward-WT	GGATTTTATGCTACATGGAACAC**GC**	61.01	44	Wild-type allele amplicon (371 bp)
Reverse-Common	TATGCCATGCCCTTTTGCCTA	60.06	47.62	—
Forward-MUT	GGATTTTATGCTACATGGAACAC**GA**	59.70	40	Mutant allele amplicon (370 bp)
Reverse-Common	TATGCCATGCCCTTTTGCCTA	60.06	47.62	—

The mismatched bases at the 3′ terminus and penultimate site of allele-specific primers (Forward-WT and Forward-MUT) are shown in bold. WT = wild-type, MUT = mutant, bp = base pair.

A specific primer set consisting of forward wild-type allele-specific (Forward-WT), forward mutant allele-specific (Forward-MUT), and common reverse (Reverse-Common) primers was designed to distinguish target alleles based on a single-nucleotide polymorphism (SNP) region in the *CUBN* gene. The specificity of this assay was further enhanced by introducing wild-type and mutant residues at the 3′ terminus of the corresponding allele-specific primers.

The penultimate position of each allele-specific primer incorporated a second mismatch, where adenine was substituted with guanine. This mismatch reduces heteroduplex stability between the primer and the non-target allele, thereby reducing non-specific amplification [24]. This modification exploits the destabilizing properties of mismatched nucleotides to enhance allele discrimination [25]. The destabilization of primer–template pairs is nucleotide-pair specific, and optimal specificity requires a balance between mismatches at the 3′ terminus and the penultimate site [24].

**Figure 1:**
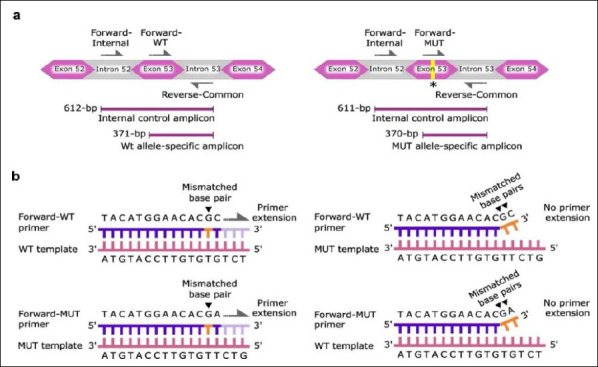
Schematic representation of primer design for single nucleotide polymorphism (SNP) genotyping of the *CUBN* gene. (a) The left panel illustrates a primer set that amplifies a 371-bp wild-type allele-specific amplicon (Forward-WT and Reverse-Common) and a 612-bp internal control amplicon (Forward-Internal and Reverse-Common). The right panel illustrates a primer set that amplifies a 370-bp mutant allele-specific amplicon (Forward-MUT and Reverse-Common) and a 611-bp internal control amplicon (Forward-Internal and Reverse-Common). The asterisk (*) denotes the position of a single cytosine deletion at the single nucleotide polymorphism site. The gray half-arrows represent the orientation of primers designed to amplify the SNP region and the conserved region distinct from the SNP. (b) The left panel illustrates forward allele-specific primers, which are perfectly aligned to their target SNP at the 3′ terminus and incorporate a single mismatched base pair at the penultimate site. This design enables efficient target allele extension and amplification. The right panel illustrates forward allele-specific primers incorporating two consecutive mismatches against their non-target alleles, one at the penultimate site and one at the 3′ terminus. These mismatches provide a stronger destabilizing effect and reduce the primer extension of non-target alleles. Black arrowheads denote mismatched base pairs. The gray half-arrows indicate the primer extension direction.

To achieve high reaction specificity, allele-specific primers were designed based on established *CUBN* mutation profiles. The Forward-WT primer included a 3′-C base for wild-type amplification, whereas the Forward-MUT primer included a 3′-A base for mutant amplification. When paired with non-target templates, Forward-MUT generated an A/G mismatch and Forward-WT generated a C/T mismatch at the 3′ terminus, producing maximal destabilization. Accordingly, a “G” base was introduced at the penultimate position to generate a weak destabilizing interaction with a “T” base in non-target templates [24]. This design ensured one mismatch against target alleles and two consecutive mismatches against non-target alleles (Figure 1b).

Schematic representation of primer design for single nucleotide polymorphism (SNP) genotyping of the *CUBN* gene. (a) The left panel illustrates a primer set that amplifies a 371-bp wild-type allele-specific amplicon (Forward-WT and Reverse-Common) and a 612-bp internal control amplicon (Forward-Internal and Reverse-Common). The right panel illustrates a primer set that amplifies a 370-bp mutant allele-specific amplicon (Forward-MUT and Reverse-Common) and a 611-bp internal control amplicon (Forward-Internal and Reverse-Common). The asterisk (*) denotes the position of a single cytosine deletion at the single nucleotide polymorphism site. The gray half-arrows represent the orientation of primers designed to amplify the SNP region and the conserved region distinct from the SNP. (b) The left panel illustrates forward allele-specific primers, which are perfectly aligned to their target SNP at the 3′ terminus and incorporate a single mismatched base pair at the penultimate site. This design enables efficient target allele extension and amplification. The right panel illustrates forward allele-specific primers incorporating two consecutive mismatches against their non-target alleles, one at the penultimate site and one at the 3′ terminus. These mismatches provide a stronger destabilizing effect and reduce the primer extension of non-target alleles. Black arrowheads denote mismatched base pairs. The gray half-arrows indicate the primer extension direction.

To further improve assay reliability, a forward-internal control primer (Forward-Internal) was integrated with the common reverse primer to ensure consistent amplification of an internal control fragment across all samples. Following optimization, the assay enabled co-amplification of a 371-bp wild-type allele fragment, a 370-bp mutant allele fragment, and a 611–612-bp internal control fragment (Figure 1a).

The *CUBN* locus of each sample was genotyped simultaneously in two separate reaction tubes. The first tube detected the wild-type allele and contained Platinum® Taq PCR buffer (Invitrogen, Carlsbad, CA, USA), dNTPs (Biotechrabbit, Berlin, Germany), MgCl^2^ (Invitrogen), Forward-Internal, Forward-WT, and Reverse-Common primers (Biobasic Inc.), Platinum® Taq DNA Polymerase (Invitrogen), and 50–80 ng genomic DNA in a 25 µL reaction volume.

The second reaction selectively amplified the mutant allele and contained identical reagents, except Forward-WT was replaced with Forward-MUT (Table 3). Amplification was performed using a MiniAmp Plus Thermal Cycler (Thermo Fisher Scientific, Waltham, MA, USA) with standardized cycling conditions, consisting of an initial denaturation at 95C for 5 min, followed by 35 cycles of denaturation at 95°C for 30 s, primer annealing at 59°C for 2 min, and extension at 72°C for 1 min, with a final extension step at 72°C for 10 min.

**Table 3:** Composition of a 25 L PCR reaction mixture for *CUBN* genotyping.

Reagent	Stock concentration	Volume added (µL)	Final concentration in 25 µL
Platinum® Taq PCR buffer	10 ×	2.5	1×
dNTP mixture	2 mM	2.5	0.2 mM each
MgCl_2_	50 mM	0.75	1.5 mM
Forward-Internal primer	10 µM	0.75	0.3 µM
Forward-WT primer*	10 µM	0.75	0.3 µM
Forward-MUT primer*	10 µM	0.75	0.3 µM
Reverse-Common primer	10 µM	0.75	0.3 µM
Platinum® Taq DNA polymerase	5 U/µL	0.1	0.5 U
Template DNA	50–80 ng/µL	1.0	50–80 ng
Nuclease-free water	–	15.9	–
Total		25.0	

* Forward-WT and Forward-MUT primers were added to a separate reaction tube.

PCR products were resolved on 1.5% agarose gels prepared in 1× TBE buffer and pre-stained with ethidium bromide (0.5 µg/mL). Electrophoresis was conducted at 100 V for approximately 30 min, and fragments were visualized under UV illumination using a Gel Doc™ EZ Documentation System (Bio-Rad Laboratories, Hercules, CA, USA).

### Synthetic mutated DNA fragments of *CUBN*

**Figure 2:**
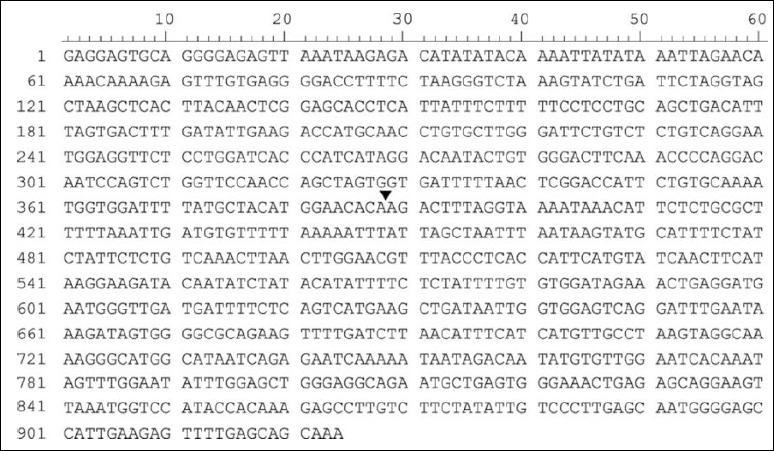
Schematic of the synthetic 924-bp DNA fragment containing the *CUBN* gene mutation locus (*CUBN*:c.8392delC). Arrowhead indicates a single cytosine deletion mutation in the target region.

Due to the absence of naturally occurring homozygous mutant individuals, a synthetic 924-bp DNA fragment containing the *CUBN* mutation locus was used as a positive control (Figure 2). The fragment was designed using GenBank accession No. NC_006584.3 and synthesized by Invitrogen Custom Gene Synthesis (GeneArt® Strings™ DNA fragments, Thermo Fisher Scientific, Waltham, MA, USA).

Schematic of the synthetic 924-bp DNA fragment containing the *CUBN* gene mutation locus (*CUBN*:c.8392delC). Arrowhead indicates a single cytosine deletion mutation in the target region.

### Sequencing

Following PCR-based *CUBN* genotyping, all 107 samples were further analyzed by Sanger sequencing. Target sequences were amplified using Forward-Internal and Reverse-Common primers. PCR products were purified using the NucleoSpin® Gel and PCR Clean-up Kit (Macherey-Nagel, Düren, Germany) and sequenced by Macrogen Inc. (Seoul, Republic of Korea).

Consensus sequences were generated using SnapGene® Viewer (GSL Biotech LLC, San Diego, CA, USA) and aligned with the reference *CUBN* sequence in the NCBI database. Sequencing chromatograms were evaluated using Phred quality scores, retaining regions with scores ≥ 20 and validating variants with scores ≥ 30.

### Statistical analysis

Prevalence and allele frequencies were calculated as proportions, and 95% confidence intervals (CI) were estimated using the Wilson score method to improve accuracy for rare events in moderate sample sizes.

## RESULTS

### Technical validation of combined double-mismatch allele-specific and multiplex PCR assays

All 107 samples were successfully and reliably genotyped for the *CUBN* gene using multiplex PCR integrated with double-mismatch allele-specific primers. This approach enabled discrimination of *CUBN* genotypes based on three distinct DNA fragment patterns: homozygous wild-type (WT; *CUBN* (+/+)), heterozygous carrier (*CUBN* (+/−)), and homozygous mutant (*CUBN* (−/−)). Samples with the *CUBN* (+/+) genotype produced a 371-bp wild-type allele-specific amplicon together with a 612-bp internal control amplicon. In contrast, the *CUBN* (+/−) genotype yielded three fragments, consisting of a 370-bp mutant allele-specific amplicon, a 371-bp wild-type allele-specific amplicon, and an internal control amplicon measuring either 611 or 612 bp.

**Figure 3:**
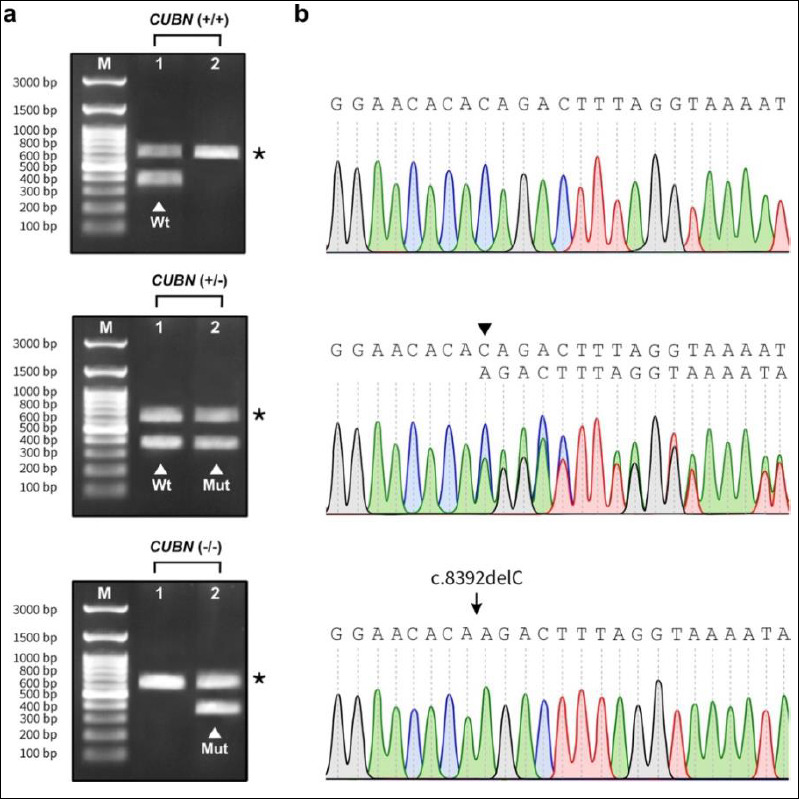
Validation of *CUBN* genotypes using integrated double-mismatch allele-specific primers with multiplex polymerase chain reaction assays, confirmed by DNA sequencing. (a) Representative agarose gel images show the amplicon pattern corresponding to the *CUBN* (+/+) (upper), *CUBN* (+/−) (middle), and *CUBN* (−/−) (lower) genotypes. The white arrowheads indicate 371-bp wild-type allele-specific (Wt) amplicons in Lane 1 and 370-bp mutant allele-specific amplicons (Mut) in Lane 2. The asterisks (*) denote the 611- or 612-bp internal control amplicons present in both lanes. M, 100-bp DNA marker; Lane 1, wild-type allele-specific amplicon and internal control amplicon; Lane 2, mutant allele-specific amplicon and internal control amplicon. (b) Sanger sequencing chromatograms illustrating the three *CUBN* genotypes. M, 100-bp DNA marker. The *CUBN* (+/+) individual (upper panel) exhibited continuous and uniform peaks, confirming the absence of mutation. In the *CUBN* (+/−) individual (middle panel), the black arrowhead indicates the overlapping sequence traces at the mutation site, indicating the presence of both alleles. The arrow in the synthetic 924-bp mutated DNA fragment (lower panel) denotes a single cytosine deletion mutation locus (c.8392delC).

The assay performance was further validated using synthetic mutated DNA fragments. As expected, this positive control generated a *CUBN* (−/−) genotype pattern characterized by a 370-bp mutant allele-specific amplicon and a 611-bp internal control amplicon (Figure 3a). PCR amplification of both allele-specific and internal control fragments produced distinct, sharp bands of the expected sizes on agarose gels. All amplicons showed clear resolution with minimal background or nonspecific amplification, allowing unambiguous discrimination between wild-type and mutant alleles.

Validation of *CUBN* genotypes using integrated double-mismatch allele-specific primers with multiplex polymerase chain reaction assays, confirmed by DNA sequencing. (a) Representative agarose gel images show the amplicon pattern

The multiplex double-mismatch allele-specific PCR assay demonstrated high reproducibility across all replicates, with 100% concordance observed. Genotyping results for all 107 samples were fully consistent with those obtained by Sanger sequencing, with no false-positive or false-negative findings detected (Figure 3b). Collectively, these results confirm that the proposed assay is highly efficient, sensitive, and specific, providing a reliable detection platform for the targeted *CUBN* mutation.

### *CUBN* genotype profiles associated with IGS

The genotypic distribution and allele frequencies of the *CUBN* gene among the 107 Border Collies analyzed are summarized in Table 4. Of these, 106 dogs were identified as homozygous WT (*CUBN* (+/+)), corresponding to 99.1% of the population (95% CI: 94.8%–99.8%). Only one individual carried the heterozygous genotype (*CUBN* (+/−)), representing 0.9% (95% CI: 0.16%–5.0%). No dogs were found to harbor the homozygous mutant genotype (*CUBN* (−/−)) (0%; 95% CI: 0%–4.3%).

**Table 4:** Distribution of *CUBN* genotype and allele frequencies in 107 Border Collies.

Category	Genotype/Allele	Frequency (%)	Wilson 95% CI
Genotype frequencies	*CUBN* (+/+) (n = 106)	99.1	94.8–99.8
	*CUBN* (+/−) (n = 1)	0.9	0.16–5.0
	*CUBN* (−/−) (n = 0)	0	0–4.3
Allelic frequencies	Wild-type allele	99.6	98.2–99.9
	Mutant allele	0.4	0.07–1.8

The calculated allele frequencies indicated a predominance of the wild-type allele at 99.6% (95% CI: 98.2%–99.9%), whereas the mutant allele frequency was estimated at 0.4% (95% CI: 0.07%–1.8%). Taken together, the absence of homozygous mutant individuals and the very low mutant allele frequency suggest that the estimated prevalence of IGS is relatively low in this Border Collie population.

## DISCUSSION

### Development and validation of the genotyping approach

This study aimed to investigate *CUBN* variants associated with IGS in Thai Border Collies by integrating double-mismatch allele-specific primers with a multiplex PCR assay. Allele-specific primers were deliberately designed with an artificial nucleotide mismatch at the penultimate position to enhance discrimination among closely related SNP variants. The incorporation of double-mismatch allele-specific primers into a multiplex PCR format further improved assay specificity and reduced the likelihood of false-negative results.

A forward-internal control primer was designed to target a conserved region within intron 52 without nucleotide mismatches. The inclusion of parallel internal control amplification provided an additional reliability feature that is not commonly incorporated in previously published allele-specific PCR designs for IGS. When paired with the common reverse primer, this internal control primer consistently amplified a fragment of 611- or 612-bp across the SNP region in all samples. The internal control fragment was clearly visualized and readily distinguished from the smaller allele-specific products (370-bp for the mutant allele and 371-bp for the WT allele) by agarose gel electrophoresis (Figure 3a).

To further validate assay performance, a custom-designed 924-bp synthetic DNA fragment representing the mutant allele was included to confirm the detection of homozygous mutant genotypes. This strategy represents a novel control approach that enables reliable interpretation of results in populations in which homozygous mutants are extremely rare. This is the first study to adapt and validate a double-mismatch allele-specific PCR assay for the rapid and cost-efficient detection of *CUBN* mutations in Thai clinical and breeding settings. Compared with Sanger sequencing or real-time PCR, the proposed assay offers an economical and scalable workflow suitable for resource-limited veterinary laboratories in Thailand.

### Practical implications for genetic screening and disease control

The findings of this study have important practical implications for genetic counselors, breeders, and clinicians. Integration of these results into genetic counseling frameworks allows collaboration with breeders to develop long-term preventive breeding strategies that maintain disease-free populations while preserving genetic diversity. Routine genetic screening before breeding can help avoid carrier-to-carrier matings and prevent the production of affected offspring. In addition, this diagnostic approach provides clinicians with a practical tool for incorporating genetic testing into routine diagnostic protocols for dogs presenting compatible clinical signs or originating from high-risk lineages.

### Population structure and *CUBN* genotype distribution

A genetic survey was conducted on 107 Border Collies in Thailand, comprising 33 privately owned dogs with unrelated parental origins and 74 dogs from seven breeding kennels. Kennel A contributed the largest proportion of dogs (n = 49), with approximately one-third sharing common parentage, whereas kennels B (n = 5), C (n = 8), D (n = 4), E (n = 2), F (n = 2), and G (n = 4) contributed smaller numbers. Pedigree verification demonstrated that the study population represented a heterogeneous genetic pool. Nearly half of the dogs were direct descendants of Border Collies originating from the United States, while the remaining dogs traced ancestry to Australia, Russia, Austria, France, Hungary, Italy, and the United Kingdom.

Genotypic analysis revealed a predominance of the WT *CUBN* genotype. Only one dog was identified as a heterozygous carrier, and no dogs were homozygous for the targeted *CUBN* mutation. The estimated WT and mutant allele frequencies were 99.6% and 0.4%, respectively. The carrier dog was a privately owned, neutered female that was clinically healthy based on physical examination and owner-reported history. No signs suggestive of cobalamin deficiency or IGS-like disease were observed, although hematological and biochemical testing was not performed. Complete pedigree information was unavailable for this adopted dog, limiting the ability to trace its ancestral origin.

### Comparison with international studies and population genetics considerations

A cohort study from Switzerland involving 203 Border Collies reported a substantially higher carrier frequency of 6.2% for the *CUBN* mutation [20], whereas a large-scale study in Japan involving 500 dogs identified a carrier frequency of 3% and a mutant allele frequency of 1.5% [23]. Despite differences in sample size and population structure, these findings highlight marked geographic variation in *CUBN* mutation distribution among Border Collie populations. The higher carrier frequency in Switzerland [20] may reflect a relatively closed gene pool with restricted gene flow, promoting the accumulation of deleterious alleles over generations. In contrast, the intermediate frequency reported in Japan [23] likely reflects differences in breeding practices and population structure, where moderate genetic diversity and founder expansion may partially dilute deleterious allele accumulation.

Analysis of imported and locally bred Border Collies in Thailand underscores the influence of international breeding lines on the distribution of hereditary disorders in emerging populations. The relatively low carrier frequency observed in this cohort is likely attributable to a geographically diverse population structure composed of dogs imported from multiple countries and their descendants. Such diversity establishes a heterogeneous founder population, mitigating founder effects and reducing *CUBN* mutation risk.

### Study limitations and future perspectives

Despite the predominance of the WT *CUBN* genotype, several limitations should be considered. First, the relatively small sample size (n = 107) limits the power to detect rare genotypes, particularly heterozygous and homozygous mutant states. This constraint is largely due to the absence of a centralized registry for Thai Border Collies, restricting access to a well-documented population. Limited awareness of breed-predisposing diseases, particularly IGS, may also have reduced owner and breeder participation.

Second, the use of convenience sampling rather than randomized selection may have resulted in uneven representation of specific kennels, introducing potential selection bias and limiting generalizability to the broader Thai Border Collie population. Third, pedigree analysis was not performed because comprehensive multigenerational lineage records were unavailable for many dogs, particularly privately owned and adopted individuals. Consequently, future studies incorporating larger sample sizes, well-documented pedigrees, and stratified random sampling are required to refine prevalence estimates and better characterize transgenerational inheritance patterns of IGS.

### Integration with existing genetic screening data

This study complements recent genetic screening efforts targeting multidrug resistance 1 (*MDR1*) [26], non-homologous end-joining factor 1 (*NHEJ1*) [27], and vacuolar protein sorting 13 homolog B (*VPS13B*) [28] in Thai Border Collies conducted between 2021 and 2023. These studies reported a complete absence of *MDR1* mutations, a 15.6% carrier frequency, and 7.8% mutant allele frequency for *NHEJ1* associated with Collie eye anomaly (n = 45), and carrier and mutant allele frequencies of 4% and 2% for *VPS13B* associated with trapped neutrophil syndrome (n = 100). Although detection of *NHEJ1*, *VPS13B*, and *CUBN* mutations indicates potential hereditary disease transmission, the very low prevalence of *CUBN* carrier and homozygous mutant genotypes suggests a generally favorable genetic health profile for Thai Border Collies at these loci.

## CONCLUSION

This study successfully identified *CUBN* variants associated with IGS in Thai Border Collies using a double-mismatch allele-specific multiplex PCR assay. Among 107 dogs analyzed, 106 individuals (99.1%) were homozygous WT, one dog (0.9%) was a heterozygous carrier, and no homozygous mutant individuals were detected. The estimated WT and mutant allele frequencies were 99.6% and 0.4%, respectively, indicating a very low prevalence of the *CUBN* mutation in this population. All PCR-based genotyping results showed complete concordance with Sanger sequencing, confirming the robustness and accuracy of the assay.

The validated assay provides a rapid, reliable, and cost-efficient diagnostic tool for routine *CUBN* genotyping in clinical and breeding settings. Its scalability and low resource requirement make it particularly suitable for veterinary laboratories with limited access to sequencing platforms. Integration of this assay into genetic counseling programs can assist breeders in implementing preventive mating strategies, avoiding carrier-to-carrier pairings, and maintaining disease-free populations while preserving genetic diversity. Clinicians may also apply this method as part of diagnostic workflows for dogs presenting compatible clinical signs or originating from high-risk lineages.Major strengths include the use of a novel double-mismatch allele-specific multiplex PCR design with an internal control amplicon, ensuring high specificity and minimizing false-negative results. The inclusion of a synthetic mutant control enabled validation of homozygous mutant detection despite the rarity of such genotypes. In addition, complete agreement with Sanger sequencing supports the analytical validity of the assay.

Several limitations should be acknowledged. The moderate sample size may limit the detection of rare genotypes and reduce statistical power. Convenience sampling and uneven representation among kennels may introduce selection bias, restricting extrapolation to the entire Thai Border Collie population. Furthermore, incomplete pedigree information for many dogs prevented detailed transgenerational inheritance analysis.

Future studies should incorporate larger, randomly sampled cohorts with comprehensive pedigree data to refine prevalence estimates and better characterize population-level inheritance patterns of IGS. Integration of this PCR-based approach with high-throughput genotyping platforms may further enhance large-scale surveillance. Longitudinal studies combining genetic data with clinical and biochemical assessments would also improve understanding of genotype–phenotype relationships.

Overall, this study provides the first population-level insight into *CUBN*-associated IGS in Thai Border Collies and demonstrates that the mutation occurs at a very low frequency in this genetically diverse population. The validated assay offers a practical foundation for routine genetic screening and supports evidence-based breeding and disease prevention strategies, contributing to improved genetic health management of Border Collies in Thailand.

## DATA AVAILABILITY

Additional data supporting the findings of this study are available from the corresponding author upon reasonable request.

## AUTHORS’ CONTRIBUTIONS

CL: Examined the clinical condition of the animals, collected blood samples, designed the study, performed the experiments, analyzed data, drafted, reviewed, and edited the manuscript. NA: designed the study, analyzed data, reviewed and edited the manuscript. NP: Provided technical laboratory support, reviewed and edited the manuscript.

## COMPETING INTERESTS

The authors declare that they have no competing interests.

## PUBLISHER’S NOTE

Veterinary World remains neutral with regard to jurisdictional claims in the published institutional affiliations.
